# Applications of chitosan in oral health and diseases

**DOI:** 10.3389/froh.2025.1632233

**Published:** 2025-10-07

**Authors:** Balen Hamid Qadir, Mohammed Khalid Mahmood, Tara Ali Rasheed, Kawan Salah Othman, Mohammed Abdalla Mahmood, Mohammed Aso Abdulghafor, Handren Ameer Kurda, Zana Fuad Noori, Botan Barzan Tahir, Mohammed Taib Fatih, Hevi Nihad Mohammed Fadhil, Vincent Romao, Arthur Falguiere, Romain Lan

**Affiliations:** 1 Department of Dentistry, Komar University of Science and Technology, Sulaymaniyah, Kurdistan, Iraq; 2 Faculty of Medical and Paramedical Sciences, French National Center of Scientific Research (CNRS), French Blood Establishment (EFS), Bio-Cultural Anthropology, Law, Ethics and Health (ADES), Aix-Marseille University, Marseille, France; 3 College of Dentistry, University of Sulaimani, Sulaimani, Kurdistan, Iraq; 4 College of Dentistry, The American University of Iraq—Sulaimani, Sulaymaniyah, Iraq; 5 Oral Surgery Department, Timone University Hospital, Marseille, Provence-Alpes-Côte d'Azur, France

**Keywords:** chitosan, dentistry, dental material, natural polysaccharide, dental health, marine drugs, oral health

## Abstract

Natural polysaccharides are polymers that are typically made up of over ten monosaccharides connected by glycosidic linkages. Because of their sustainability, renewability, biodegradability, and non-toxicity, natural polysaccharides and their derivatives have found extensive application in the food, pharmaceutical, medical and dental industries in recent years. Chitosan is one of the important members of the natural polysaccharide family with proven antimicrobial, biocompatibility, biodegradability, solubility and film-forming properties. These characteristics make chitosan an excellent candidate for biomedical applications. Chitosan, either alone or in conjunction with other materials, were used in many preclinical and clinical studies related to oral health. There is a growing body of research in these areas indicating its raising trends. The aim of this narrative review is to provide a comprehensive update on the applications of chitosan in various fields of dentistry. To investigate this, PubMed, MEDLINE, Scopus and Embase databases were searched until April 2025. A total of 41 clinical human trials published in the last ten years were included. The contents of these studies were qualitatively and critically assessed. Based on the studies covered in this review, the following conclusions can be drawn: (1) Chitosan is a safe and effective biomaterial for oral and dental applications. (2) Chitosan has been used nearly in all fields of stomatology. (3) Chitosan has been used in the form of gel, solution, scaffold, mouthwash, brush, toothpaste, coating material, intra-canal medicament, sustainable drug release compound, chewing gum, adhesive and varnish. (4) The preliminary research shows promising results. (5) However, at this stage and compared to the established treatment modalities, their utilization in human clinical trials should be viewed as promising supplementary and adjuncts pending further validation through further clinical trials.

## Introduction

Natural polysaccharides are either branched or non-branched polymers that are typically made up of over ten monosaccharides connected by glycosidic linkages. Animals, microbes, and plants all contain large amounts of these polysaccharides (1, 2). Their anti-inflammatory, antioxidant, and anti-tumor qualities support their use in biomedicine. Because of their sustainability, renewability, biodegradability, and non-toxicity, natural polysaccharides and their derivatives have found extensive application in the food, pharmaceutical, and dental industries in recent years (3, 4). Additionally, the structural diversity of natural polysaccharides is responsible for their widespread use in the aforementioned industries (5).

**Figure 1:**
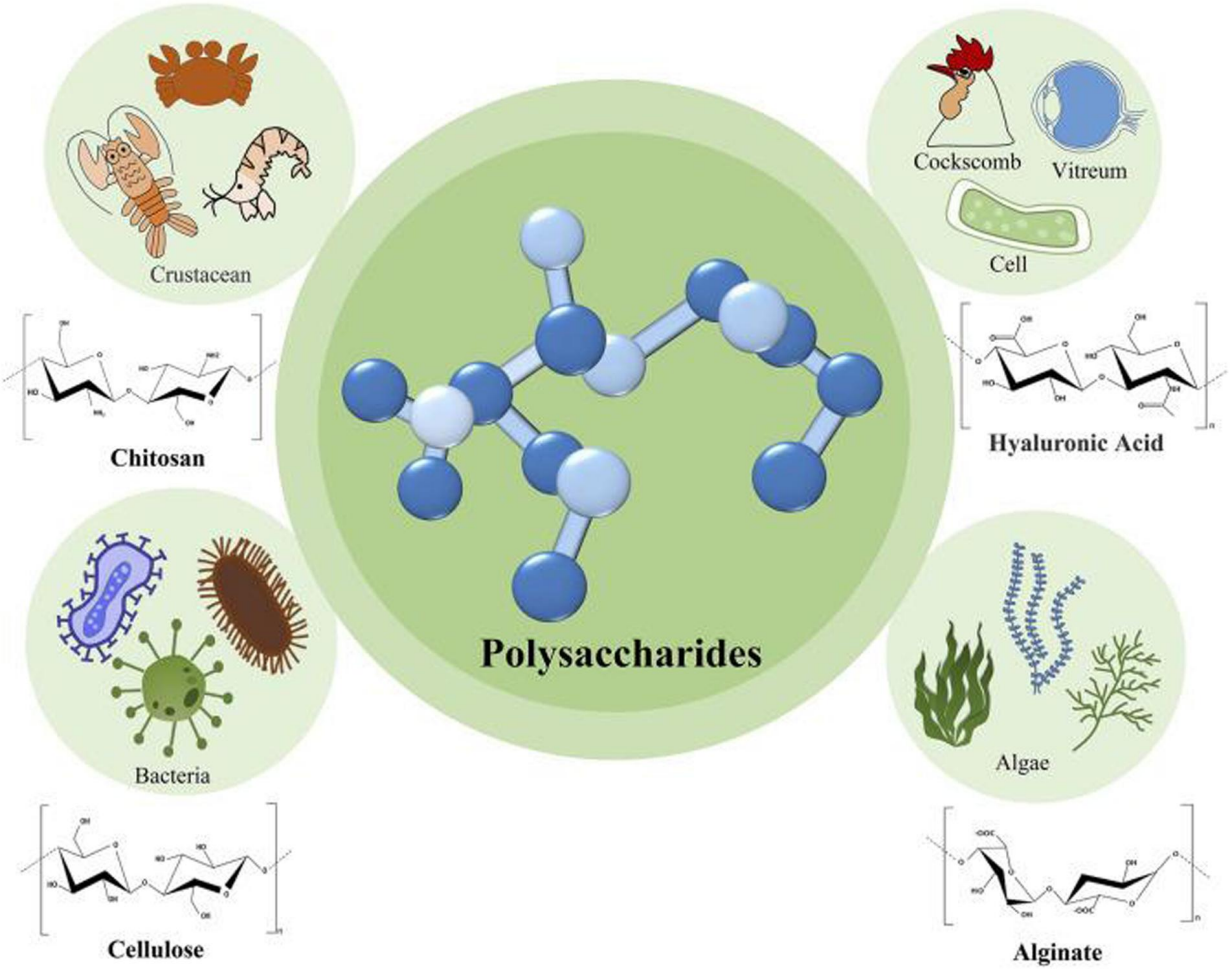
Main natural polysaccharides and their sources. Reproduced from (2).

The two main categories of polysaccharides are homogeneous and heterogeneous. Chitosan, starch and cellulose are examples of homogeneous polysaccharides, which are formed by connecting several monosaccharide molecules of the same type. Different monosaccharide molecules can be converted into heterogeneous polysaccharides (5, 6). Among the common heterogeneous polysaccharides are chondroitin sulfate and hyaluronic acid. Figure 1 shows the main natural polysaccharides and their sources.

Chitosan is a linear, semi-crystalline natural polymer that is generated from chitin and can be found in fungal and crustacean cell walls. Chitosan has been licensed for use in food and medications by the U.S. Food and Drug Administration (FDA) (2, 7).

The perfect dental and oral biomaterial should have the following features: it should be antimicrobial and biomimetic, have no immune-stimulating qualities, be readily manufactured or produced, be easily and rapidly metabolized in the case of complete integration with human body (8–12). Chitosan possesses nearly all these positive properties.

Chitosan is used in stomatology for antibacterial applications, drug delivery, target therapy and sustainable drug release systems (3, 5, 6, 11). According to studies, it successfully prevents Streptococcus mutans, which is the primary cause of dental caries, from forming biofilms and producing acid (13, 14). Chitosan has served as the model for several delivery systems that provide medications for the treatment of tissue regeneration and periodontitis (15). Chitosan has been used in oral health as an independent agent, in conjunction with other material and even as a coating material (16–18). This biocompatible material, has a wide spectrum of use in stomatology, covering a broad range from oral surgery to endodontics and even anticariogenic activities (19–21). Over time, some hydrogel and nanomaterials have been modified using chitosan and its derivatives and utilized in the treatment of oral diseases (6).

There is a large body of literature on the use of chitosan in dentistry concerning preclinical and *in vitro* study designs. Furthermore, there is a growing body of research on the use of chitosan in stomatology in clinical trials involving human subjects. However, there are few reviews on chitosan's use in clinical dentistry. In an article published in 2019, Cicciù et al. systematically reviewed the use of chitosan in dentistry in articles published between 2009 and 2019 with the inclusion of 12 records (22). More recently, Arora et al. reviewed the role of chitosan hydrogels in clinical dentistry (23). The present review provides a new perspective on the topic. Since it offers an update on the subject with an emphasis on more recent publications and with a higher number of included studies. Moreover, instead of a single material, it covers all forms of chitosan application (toothpaste, brush, gel, irrigation solution, mouthwash, etc.).

## Methodology

The research questions of the review were “how chitosan was used in oral health related clinical human studies?” and “what are the results of these studies especially when compared to standard and established treatment options?” To investigate these questions, PubMed, Embase, Scopus and MEDLINE databases were searched using relevant keywords. The following search strategy was applied: (“chitosan” OR “Chitosan”) AND (“dentistry” OR “oral health” OR “dental material” OR “oral disease” OR “dental caries” OR “periodontitis”). The following filters were used: In the last 10 years, Clinical Study, Clinical Trial, Randomized Controlled Trial, English, Humans. All the clinical human trials published in the last ten years on the usage of chitosan, either alone or in conjunction with other materials in the field of oral health and disease were included. Only articles in English language were considered for inclusion. All the preclinical, *in vitro*, animal, and *in situ* studies were excluded.

## Results

**Figure 2:**
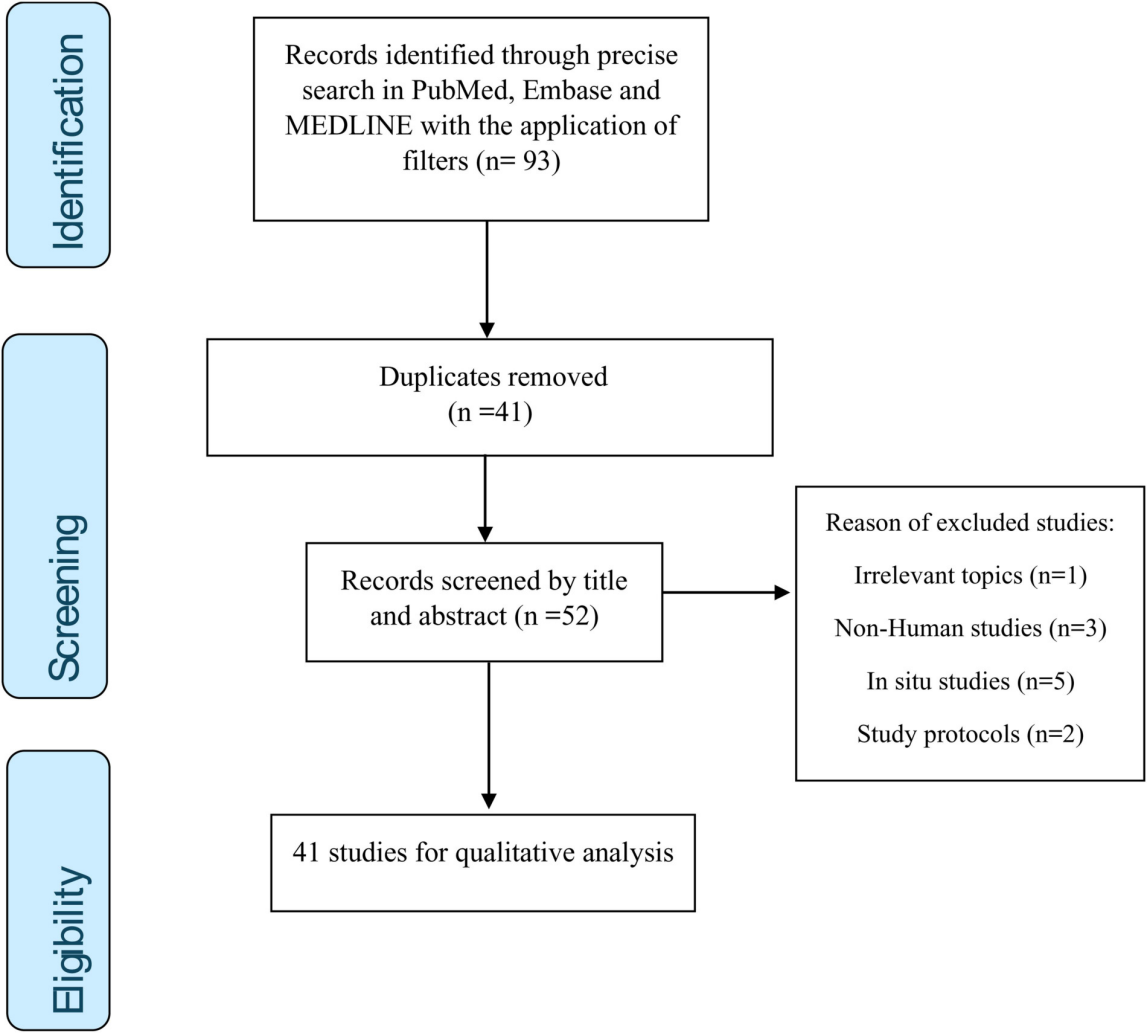
Flowchart of study selection process.

The initial search revealed 93 results. First, 41 duplicates were removed. After screening the title and abstracts, another 11 records were excluded. Reasons of these exclusions were mainly irrelevant topic, non-human studies, *in situ* studies and study protocols. Finally, a total of 41 relevant articles were included in the review. Figure 2 shows the flowchart of the study selection process. The following sections present the results according to the specific usage field of chitosan in oral health.

### Oral surgery

Nine studies used chitosan in oral surgery with various findings. Most of the applications were related to wound healing of the extraction site. The main measured outcomes were pain, hemostasis, alveolar ridge preservation, bacterial colonization and inflammation. Chitosan was chiefly used as a wound dressing material, scaffold, gel and mouthwash. All the studies used adult population as their participants, except one that investigated chitosan's effect among a pediatric age group. Some of the studies were performed on healthy populations, whereas some others included participants with special conditions like liver cirrhosis and patients on anticoagulants. Table 1 presents the main characteristics of these studies.

**Table 1:** Included studies in the field of oral surgery.

Publications	Sample size	Material	Used for	Findings	Statistical significance
		T: Test			
		C: Control			
Radhakrishna et al. (24)	54 patients undergoing tooth extractions who were on single or dual antithrombotic with an INR < 3.	T: Chitosan dressing	Hemostasis	Chitosan dressing induced hemostasis faster than cotton pressure packs (96 ± 4 vs. 797 ± 23 s; *P* < .001). Compared to cotton pressure packs, chitosan enhanced alveolar clinical healing index by 88.9%. Not one chitosan patient suffered alveolar osteitis, unlike 3.7% of cotton pressure pack patients.	Yes
		C: Conventional cotton pressure pack			
Thakkar et al. (25)	87 primary molar extraction sites from 29 participants.	Group A was given the control first, then chitosan, and finally low-level laser therapy (LLLT).	Analgesic and hemostatic efficacy of chitosan	The LLLT group had the lowest mean pain score, followed by chitosan and the control group had the highest. This difference was statistically significant. The three groups had similar postoperative hemorrhage after 15 min. Chitosan was the favorite choice of children and their parents.	Pain score: Yes
					Bleeding: No
		Group B received the laser intervention first, then chitosan, and lastly the control.			
		Group C was exposed to the chitosan intervention first, then the control, and finally the laser.			
Yadav et al. (26)	32 patients requiring free gingival graft (FGG) surgery	T: Chitosan-based dressing (CBD): 2 min pressure with wet gauze	Management of palatal donor site	At weeks 2 and 3, the test group exhibited higher complete epithelialization and VAS scores for color match, but only at week 4 was the difference statistically significant. Patients in the test group experienced significantly less acute and delayed bleeding (*p* < 0.05). Average pain scores and analgesic use were greater in 5-day assessments, but the differences were not significant.	Complete epithelialization: No.
					Color match: Yes.
					Immediate and delayed bleeding: Yes.
					Pain and analgesic use: No.
Lopez-Lopez et al. (27)	47 patients (94 molars extracted)	T1: chlorhexidine, dexpanthenol, allantoin and chitosan [Bexident Post (BP)]	Pain and inflammation	BP reduced pain intensity more than chlorhexidine (7 day mean scores 3.7 vs. 5.3; *p* = 0.0001). BP reduced inflammation 50% (6 mm vs. 12 mm; *p* = 0.0001) better than chlorhexidine. Analgesic tablet usage decreased with BP (13 vs. 24; *p* < 0.05) compared to control. 64% of BP users had “excellent” cicatrization compared to 13% of control users (*p* = 0.0001).	All parameters: Yes
		T2: chlorhexidine			
Al-Madhagy et al. (28)	12 patients (24 alveolar sockets post-extraction)	T: Electrospun chitosan/polyvinyl alcohol (CS/PVA) nanofibrous scaffolds	Preserving the alveolar ridge	Compared to the control group, testing showed a significantly lower mean vertical resorption of 1.1 mm (*P* < 0.05). The test group exhibited a considerably decreased mean horizontal bone resorption (−0.69 ± 0.41 mm) compared to the control group (−2.01 ± 1.04 mm, *P* < 0.05). Compared to the control group (448.73 ± 93.23 HU), the study group showed a substantial increase in bone density (722.03 ± 131.17 HU) after 4 months.	All parameters: Yes
		C: Secondary intention healing			
Zorilla et al. (29)	21 patients (42 extracted wisdom teeth)	T1: Chlorhexidine gel	Microbiologic colonization of the suture threads	Blood Agar and *Mitis Salivarius* Agar grew faster with chlorhexidine-chitosan than placebo (*p* = 0.04). All groups experienced similar post-surgery pain. Patients healed faster with chlorhexidine-chitosan gel than placebo (*p* = 0.03) and hyaluronic acid gel (*p* = 0.01).	Microbial colonization: Yes
		T2: chlorhexidine-chitosan gel			
					Pain: No
					Healing: Yes
		T3: hyaluronic acid gel			
		C: Neutral water-based gel			
Efeoglu et al. (30)	50 liver cirrhosis patients (80 extractions)	T1: Chitosan	Hemostasis	Patient demographics, cirrhosis classification, trauma score, and bleeding time did not differ statistically significantly across the groups.	No
		T2: Surgicel			
Sáez-Alcaide et al. (31)	36 patients with bilaterally and symmetrically impacted lower third molars	T: A gel combining 0.2% chlorhexdine, 0.5% chitosan, 5% dexpanthenol, 0.15% allantoin and 0.01% sodium saccharin.	Pain and inflammation	In alveolitis rates, the control group (13.9%) and study group (0%), were not significantly different. From day 0 to day 7, trismus and edema decreased significantly, with 22.2% of the control group experiencing “excellent” wound healing and 97.2% of the EG group (*p* < 0.001). Compared to the placebo group, the study indicated a decrease in mean VAS ratings (2.56 ± 1,19) after surgery (*p* = 0.002). Test group consumed fewer analgesic tablets (0.26 ± 0.51) in the first 92 h than control (0.56 ± 0.67) (*p* = 0.003).	Alveolitis: No
					Trismus, swelling and healing: Yes
					Mean VAS: Yes
					Analgesic use: Yes
		C: Placebo			
Kumar et al. (32)	33 patients on oral anticoagulant therapy having two or more surgical sites	T: Hemcon Dental Dressing (HDD) based on chitosan	Hemostasis	All HDD operative sites achieved hemostasis in 1.49 min and wound control in 4.06 min (*p* < 0.001). HDD-treated sites had considerably lower post-operative pain (1.87, 1.27 on 1st and 3rd days) than control sites (4.0, 1.87) (*p*-value (0.001, 0.001). HDD-treated oral surgical wounds revealed significant healing improvements in the first and third post-operative days (*p* < 0.0001).	All parameters: Yes
		C: Conventional pressure pack			

HDD, hemcon dental dressing; VAS, visual analogue scale; HU, hounsfield units; LLLT, low-level laser therapy; INR, international normalized ratio; CBD, chitosan-based dressing.

### Dental implantology

Six studies used chitosan in dental implantology. The main purpose of these studies was treatment of peri-implantitis. The common measured outcomes were bleeding on probing (BOP), bleeding index (BI), plaque index (PI), clinical attachment level (CAL), radiographic bone level and probing pocket depth (PPD). Chitosan was mainly used as brush. Table 2 presents the key characteristics of these studies.

**Table 2:** Included studies on dental implantology.

Publications	Sample size	Material	Used for	Findings	Statistical significance
		T: Test			
		C: Control			
Wohlfahrt et al. (38)	11 patients (24 dental implants) with peri-implantitis treated for 6 months.	T: chitosan brush	Treatment of peri-implantitis	Between baseline and six months, there were notable decreases in BOP in both groups. In comparison to the implants treated with titanium curettes, the test implants treated with the chitosan brush showed a greater improvement in BOP at two and four weeks.	Intragroup: Yes
					Intergroup (BOP) at 2 and 4 weeks: Yes
		C: titanium curettes.			
Koldsland et al. (39)	44 subjects with peri-implantitis	T: chitosan brush	Treatment of peri-implantitis	At the 6-month baseline assessment, a large percentage of implants were registered as inflammatory (>80% BOP in both the test and control groups), and this percentage stayed high during the monitoring period. The findings were stable across all clinical measures.	No
		C: titanium curettes.			
Mayer et al. (40)	69 patients with 106 implants, diagnosed with peri-implantitis	T: chitosan bristle, soft tissue curettage combined with application of 0.95% hypochlorite and 1 mg minocycline HCl.	Treatment of peri-implantitis	Both groups showed significant reductions in mean PI, PPD, CAL, and BOP at 6 months (0.71 ± 0.57, 0.81 ± 0.55; 4.77 ± 0.73 mm, 4.42 ± 0.5 mm; 5.03 ± 0.86 mm, 5.13 ± 0.73 mm; chitosan group had considerably superior PPD results than controls after 6 and 12 months. The chitosan group had considerably decreased bleeding at 12 months (15.3% ± 6.2 vs. 25.1% ± 8.2).	Intergroup PPD and BOP: Yes
		C: ultrasonic debridement and soft tissue curettage.			
Khan et al. (41)	31 patients with mild to moderate peri-implantitis	Test: mechanical debridement with oscillating chitosan brush (OCB).	Treatment of peri-implantitis	At 12 months, both groups had significantly lower PPD, BI, and pus. Both groups had stable radiographic bone levels at 12 months. The groups had no statistically significant differences in any metrics.	Intragroup: Yes
					Intergroup: No
		Control: titanium curette (TC).			
Khan et al. (42)	39 patients with one implant with mild to moderate peri-implantitis	T: Oscillating chitosan brush (OCB)	Treatment of peri-implantitis	Both groups experienced significant reductions in PPD and BI at six months compared to baseline (*p* < .05). PPD and BI changes were not statistically different across groups. Non-surgical peri-implantitis therapy with OCB and TC was similar.	No
		C: Titanium curettes (TC)			
Zeza et al. (43)	15 patients with mild peri-implantitis with single implants	T: Chitosan brush for professional removal of plaque	Treatment of peri-implantitis	Bone level was steady throughout the research. At every control visit, PI was nearly 0. At 2 weeks, PPD and BOP decreased considerably from baseline. Tests with steady bone level showed no bleeding in 73% of patients at 24 weeks.	Yes

BOP, bleeding on probing; PPD, periodontal pocket depth; BI, bleeding index; PI, plaque index; TC, titanium curette; OCB, oscillating chitosan brush; CAL, clinical attachment level.

### Periodontology

Five studies used chitosan in periodontology field. All the studies were related to the treatment of periodontal/gingival defects. The main measured outcomes were periodontal pocket depth (PPD), bleeding on probing (BOP), bleeding index (BI), gingival index (GI), plaque index (PI), clinical attachment loss (CAL) and vertical recession depth (VRD). Chitosan was chiefly used as brush, coating material, sustainable drug release compound and gels. All the studies used adult population as their participants. Table 3 presents the main characteristics of these studies.

**Table 3:** Included studies in the field of periodontology.

Publications	Sample size	Material	Used for	Findings	Statistical significance
		T: Test			
		C: Control			
Hayashi et al. (44)	40 patients with a pocket of 5 mm or deeper.	T: Antimicrobial photodynamic therapy (aPDT) by photosensitizer nanoparticles: indocyanine green ICG-encapsulated nanoparticles (ICG-nano/c) coated with chitosan.	Suppression of subgingival bacteria	There were no significant variations in PPD or BOP between test and control groups. Post-treatment colony counts in the test group were considerably lower than before treatment.	PPD: No
					Colony count: Yes
		C: pseudo aPDT without photosensitizer.			
Hussain et al. (45)	78 patients with periodontitis	T: Oscillating chitosan brush	Treatment of periodontal pockets	PPD and BOP decreased significantly in both groups. BOP did not differ between test and control groups after 6 months, however the test group showed a substantial improvement in PPD decrease (*P* < 0.01). In the test group, no BOP and PPD <4 mm produced a considerably better outcome (*P* ≤ 0.01).	Yes
		C: Subgingival treatment with curettes			
Mahmoud et al. (46)	150 pockets from patients suffering from moderate chronic periodontitis	G1: nano-structured DOX/chitosan	Periodontal regeneration by sustained release	Nano-structured chitosan and DOX films improved assessed metrics more than placebo and control groups.	Yes
		G2: DOX only			
		G3: placebo films			
Dhawan et al. (47)	9 patients and 22 sites with gingival recession defects	Group A: Amnion membrane	Treatment of gingival recession	Both groups showed statistically significant reduction in PI, GI, VRDD, and CAL and nonsignificant reduction in width of keratinized tissue at 3 and 6 months postoperatively.	PI, GI, VRDD, and CAL: Yes
					Width of keratinized tissue: No
		Group B: A functionally graded membrane (FGM) tailored by incorporating chitosan and nano-hydroxyapatite.			
					PD and CAL: Yes
Ali et al. (48)	24 patients having bony periodontal defects	T: Chitosan 2% gel containing free Simvastatin (SV)	Treatment of chronic periodontitis	Chitosan gels with SV microsponges had much lower PPD and CAL than those with free SV.	Yes
		C: SV microsponge			

VRDD, vertical recession defect depth; CAL, clinical attachment level; PI, plaque index; GI, gingival index; PPD, periodontal pocket depth; DOX, doxycycline; FGM, functionally graded membrane; SV, simvastatin.

### Oral medicine

Overall, seven included studies used chitosan in oral medicine and pathology. The purpose of the studies was to treat actinic cheilitis, oral mucositis, oral candidiasis, recurrent aphthous stomatitis and denture stomatitis. The main measured outcomes were clinical improvement of lesions, lesion size, pain severity, antifungal efficacy and number of *Candida Albican* colonies. Chitosan was chiefly used as hydrogel, nanogel, mouthwash and loading material to antibiotics. Table 4 presents the key characteristics of these studies.

**Table 4:** Included studies in the field of oral medicine.

Publications	Sample size	Material	Used for	Findings	Statistical significance
		T: Test			
		C: Control			
Liberato da Silva et al. (22)	49 subjects	T: Chitosan hydrogel containing 0.05% nanoencapsulated (NANO) imiquimod (IMIQ-0.05%-NANO)	Actinic cheilitis	The IMIQ-NANO-0.05% and IMIQ-5% groups exhibited significantly higher rates of clinical improvement.	Clinical improvement: Yes
		C: placebo hydrogel			
Samiraninezhad et al. (23)	60 patients with oral mucositis treated for 14 days	T1: doxepin mouthwash (DOX),	Oral mucositis	Lesions were examined using four indices: NCI, WHO, WCCNR, and VAS.	Yes
		T2: chitosan nanogel (CN)			
		T3: doxepin/chitosan nanogel (CN + DOX).			
				Three days after intervention, CN + DOX significantly reduced WHO, WCCNR, and VAS ratings more than the control. CN + DOX lowered NCI and WCCNR more than the control and CN seven days after the intervention. CN + DOX significantly reduced NCI 14 days after the intervention.	
		C: diphenhydramine + aluminum-magnesium mouthwash			
Gamil et al. (49)	80 diabetic patients with symptomatic oral candidiasis	T1: miconazole only	Oral candidiasis	Groups showed no significant difference in antifungal effectiveness (*P* > 0.05). Miconazole-loaded chitosan nanoparticles significantly improved indications and symptoms from baseline (70%) to the end of the research at 28 days (5%, *P* < 0.05). In the miconazole-loaded chitosan nanoparticles group, *Candida Albicans* colonies decreased significantly from baseline to the end of the trial at 28 days, with a *P* value < 0.000.	Antifungal efficacy: No
		T2: miconazole-loaded chitosan nanoparticles.			Clinical improvement: Yes
					Colony number: Yes
Samiraninezhad et al. (50)	60 patients with recurrent aphthous stomatitis	T: topical Chitosan Nanogel/Probiotic mixture (CNP).	Recurrent aphthous stomatitis	The intervention significantly reduced pain severity and lesion size in both groups. After one week, the probiotic group reduced lesion size more than the control group (*P* = 0.01). The probiotic group reduced pain severity more than the control group (*P* = 0.04).	Lesion size: Yes
					Pain severity: Yes
		C: oral rinse, a mixture of 60 ml diphenhydramine, and 60 ml aluminum-magnesium mg (ADIGEL-S).			
Abou-Taleb et al. (51)	48 patients with denture stomatitis	T1&T2: miconazole nitrate was dissolved in the plasticizer propylene glycol.	Denture stomatitis	T3 was more successful than films T1 & T2 and commercial oral gel in terms of pain assessment at three days (*P* < 0.05) and colony count at ten days (*P* ≤ 0.05). Fungi inhibition zones differed significantly (*P* ≤ 0.05) when tested with the same quantity of miconazole per agar well.	Pain score: Yes
					Colony count: Yes
					Fungi inhibition zones: Yes
		T3: miconazole nitrate-loaded with chitosan nanoparticles.			
Mustafa et al. (52)	30 patients with denture stomatitis	T: Alcohol-free chitosan-curcuminoid Mouthwash (CHI-CUR)	Denture stomatitis	After a 2-week treatment period, 80% of CHI-CUR mouthwash users experienced a full response, compared to 30% of CHX mouthwash users (*p* < 0.05). Their anticandidal effects were similar.	Erythematous lesions: Yes
					Candida colonies: No
		T: Chlorhexidine (CHX) mouthwash			
Atai et al. (53)	40 patients diagnosed with denture stomatitis	Treatment with chitosan or nystatin for 2 weeks.	Treatment of denture stomatitis	The erythematous surface area, burning sensation, time needed for clinical improvement, and quantity of blastospores and mycelia were all markedly reduced by chitosan solution.	Yes

CHX, chlorhexidine; CHI-CUR, chitosan-curcuminoid; DOX, doxepin; NCI, national cancer institute; WHO, world health organization; WCCNR, world conference on clinical and research in nursing; VAS, visual analog scale.

### Restorative dentistry

Five studies used chitosan in restorative dentistry. The purpose of the studies was assessment of restorative material, treatment of periapical changes, endodontic treatment and retreatment of failed endodontic therapy. The common measured outcomes were antibacterial activity, bacterial count, biofilm inhibition percentage, compressive and flexure length of restorative material, healing percentage, post-operative pain and regaining sensitivity. Chitosan was mainly used as restorative material, intra-canal scaffold, intra-canal medicament and intra-canal irrigation solution. Table 5 presents the key characteristics of these studies.

**Table 5:** Included studies in restorative dentistry.

Publications	Sample size	Material	Used for	Findings	Statistical significance
		T: Test			
		C: Control			
Mishra et al. (54)	50 children (7–12) years	C: conventional GIC.	*in vivo* assessment	The increase in antibacterial activity (Group T1 > T2 > C) (*P* < 0.001) and marked increase in compressive and flexure strength (Group T1 > C > T2) were observed.	Yes
		T1: chitosan modified GIC (GIC) (10% v/v)			
		T2: chlorhexidine-cetrimide (CHX-CT) modified GIC (2.5/2.5% w/w)			
Alshahhoud et al. (55)	30 teeth from 24 participants, single-rooted teeth with periapical lesions	Group A: Blood clot (BC) scaffold	Treatment of periapical change	The EMC + BC scaffold healed pulp better than the NCS + BC and BC groups after six months. At one, three, and twelve months, there were no significant differences. In addition, EMC + BC and NCS + BC had greater tooth sensitivity.	No
		Group B: a combination of Native Chitosan and blood clot (NCS + BC) scaffold.			
		Group C: Enzymatically-Modified Chitosan and Blood Clot (EMC + BC) scaffold			
Arafa et al. (56)	55 Single-rooted teeth with failed endodontic therapy.	T1: Ciprofloxacin hydrochloride (CIP) encapsulated in PLGA nanoparticles coated with chitosan (CIP-CS-PLGA-NPs).	Retreatment of failed endodontic therapy	The cumulative release of T1 and T2 after 72 h was 50.03% ± 0.7345 and 77.98% ± 3.122 respectively. Healing percentage, bacterial count and biofilm inhibition percentage were significant between the groups.	Healing percentage: Yes
					Bacterial count: Yes
					Biofilm inhibition percentage: Yes
		T2: free CIP incorporated in Pluronic® 407/188.			
Nasr et al. (57)	60 patients with necrotic mandibular premolars	T1: 3% Chitosan Nanoparticles (CNPs) T2: 2% chlorhexidine (CHX)	Antimicrobial effectiveness and post-operative pain after single-visit endodontic treatment.	The effectiveness of CNPs and CHX/CNPs against anaerobic bacteria was not significantly different from CHX or NaOCl. All irrigants affected aerobic bacteria identically. CNPs and CHX/CNPs greatly reduced post-operative pain in the first 24 h.	Antimicrobial activity: No
					Post-operative pain: Yes
		T3: CHX/CNPs			
		C: 5.25% Sodium hypochlorite (NaOCl)			
Abielhassan et al. (58)	45 patients having necrotic mature anterior teeth with periapical lesions	T1: final disinfection using low power diode LASER.	Root canal irrigation	In the first 24 h, CNPs and CHX/CNPs dramatically reduced post-operative pain. All irrigants inhibited aerobic bacteria equally.	No
		T2: Final rinse using 0.2% nano chitosan irrigation			
		C: 1.25% sodium hypochlorite irrigating solution and 17% EDTA only.			

CIP, ciprofloxacin hydrochloride; CNP, chitosan nanoparticles; GIC, glass ionomer cement; EDTA, ethylenediaminetetraacetic acid; PLGA, poly (lactic-co-glycolic acid); EMC, enzymatically-modified chitosan; NaOCL, sodium hypochlorite.

### Cariology and oral care

Five studies used chitosan in the field of cariology and oral care. The purpose of the studies was caries prevention, caries arrest and caries treatment. The main measured outcomes were extension of enamel and dentinal caries, salivary pH, cariogenic bacterial count (especially *S. Mutans*) and remineralization capacity. Chitosan was chiefly used as an active compound in toothpaste, chewing gum and liquid solution for dentinal caries. All the studies were conducted in children except one that included young adults. Table 6 presents the main characteristics of these studies.

**Table 6:** Included studies in the field of cariology and oral care.

Author	Sample size	Material	Used for	Findings	Statistical significance
Campus et al. (33)	610 children (4–5 and 6–7 years)	Four toothpastes were administered for 24 months: two with fluoride-substituted hydroxyapatite (HAF1000 and HAF1450; 1,000 and 1,450 ppmF) and magnesium-, strontium-, and carbonate-substituted in a chitosan matrix, and two with monofluorophosphate fluoridation.	Prevention of dental caries	All groups' minimum pH increased statistically, but HAF1000 and HAF1450 increased the most. Both HAF groups had considerably lower main cariogenic bacteria than fluoride groups at trial's end.	pH value: Yes
					Cariogenic bacteria: Yes
Santos et al. (34)	80 teeth in children (aged 7–9 years) with active carious lesions and dentin cavitation located on the occlusal surface of deciduous molars.	Four groups based on the caries eradication technique: Er: YAG laser (250 mJ/4 Hz) or surface therapy for burs and dentin: 2.5% distilled water or chitosan solution.	Dentin treatment after selective removal of caries	Chitosan treatment of dentin reduced *Streptococcus mutans* in both removal procedures (*p* = 0.0424). No significant changes were found in retention and secondary caries criteria over time (*p* > 0.05).	Streptococcus mutans: Yes
					Filling retention and secondary caries: No
Cocco et al. (35)	610 children (4–5 and 6–7 years old)	Four toothpastes were administered for 24 months: two with fluoride-substituted hydroxyapatite (HAF1000 and HAF1450; 1,000 and 1,450 ppmF) and magnesium-, strontium-, and carbonate-substituted in a chitosan matrix, and two with monofluorophosphate fluoridation.	Remineralization effect	HAF vs. conventional fluoridated had a significant difference in primary dentition status (active at baseline and inactive at follow-up) (*P* = 0.04). Both groups had equal percentages of inactive dentinal lesions filled at follow-up (*P* = 0.08). The NaMFP group had fewer inactivated lesions than the HAF group.	Caries activity: Yes
					Dentinal lesions: No
Cagetti et al. (36)	610 children (4–5 and 6–7 years)	Four toothpastes were administered for 24 months: two with fluoride-substituted hydroxyapatite (HAF1000 and HAF1450; 1,000 and 1,450 ppmF) and magnesium-, strontium-, and carbonate-substituted in a chitosan matrix, and two with monofluorophosphate fluoridation.	Caries preventive and caries arrest efficacy	When compared to children who received conventional toothpastes, children who received HAF toothpaste in primary dentition exhibited RRs of 39% (Gyoung) and 38% (Gold). In children treated with HAF toothpaste, the RR in the permanent teeth was 29%.	Yes
Khamverdi et al. (37)	36 subjects (18–23 years)	T: chitosan chewing gum	Salivary S. mutans counts and salivary pH.	Two groups had significantly different numbers of salivary S. mutans colonies (*p* < .001). Intervention group salivary pH mean value was not significantly different from control group (*p* = .17). This study found that chitosan chewing gum reduced salivary S. mutans colonies but did not increase salivary pH.	Salivary S. mutans: Yes
		C: placebo chewing gum			Mean salivary pH: No

HAF, hydroxyapatite-fluoride; ErYAG, erbium-doped yttrium aluminium garnet; RR, risk ratio.

### Orthodontics

Four studies used chitosan in the field of orthodontics. The main purpose of the studies was prevention of white spots and dental caries around brackets, to reduce bacterial activity and decrease inflammation after placement of orthodontic mini screws. The common measured outcomes were bacterial count and plaque pH. Chitosan was mainly used as mouthwash, varnish and bonding agent. Table 7 presents the key characteristics of these studies.

**Table 7:** Included studies in orthodontics.

Author	Sample size	Material	Used for	Findings	Statistical significance
		T: Test			
		C: Control			
Farzanegan et al. (59)	24 candidates for fixed orthodontic treatment	T: bracket was bonded conventional adhesive containing 1% chitosan NPs and 1% TiO_2_NPs.	Prevention of white spot lesions and caries.	Significant decrease in the experimental group's *S. Mutans* numbers at one day, two months, and six months in comparison to the controls.	Yes
		C: conventional orthodontic adhesive.			
Poornima et al. (60)	20 subjects (200 teeth)	T: Chitosan-based varnish C: chlorhexidine-fluoride varnish for brackets	Bacterial count and plaque pH	Chitosan-based varnish scored 98% and 95% of teeth as well as chlorhexidine-fluoride varnish after 6 weeks (*P* > 0.05). Both varnishes did not affect neutral plaque pH.	No
Fakhri et al. (61)	45 orthodontic patients	Mouthwashes are used twice a day for 1 month.	Reduction of salivary S. Mutans	All mouthwashes reduced salivary S. mutans, but CCAgNPF and chlorhexidine were equally effective (*P* > 0.05). The optimal outcomes were reached with CCAgNPF and chlorhexidine mouthwashes (*P*-value < 0.05).	T3: Yes
		T1: colloidal chitosan-silver nanoparticles-fluoride nanocomposite (CCAgNPF)			
		T2: chlorhexidine 0.2%			
		T3: combination of the two			
Hasriati et al. (62)	30 patients with orthodontic miniscrews	T1: 1% chitosan	Reducing inflammation after orthodontic miniscrews.	Chitosan and chlorhexidine proved effective in lowering bacterial count surrounding orthodontic miniscrews (*p* < 0.05). Antibacterial activity of chitosan and chlorhexidine was not substantially different (*p* ≥ 0.05) for total bacteria. The best chitosan antibacterial effect on red-complex bacteria was 58% in Tannerella Denticola.	Intragroup: Yes
		T2: 0.2% chlorhexidine digluconate			Intergroup: No
		T3: Aquadest			

NP, nanoparticle; TiO2NPs, titanium oxide nanoparticle; CCAgNPF, colloidal chitosan-silver nanoparticles-fluoride nanocomposite.

## Discussion

**Figure 3:**
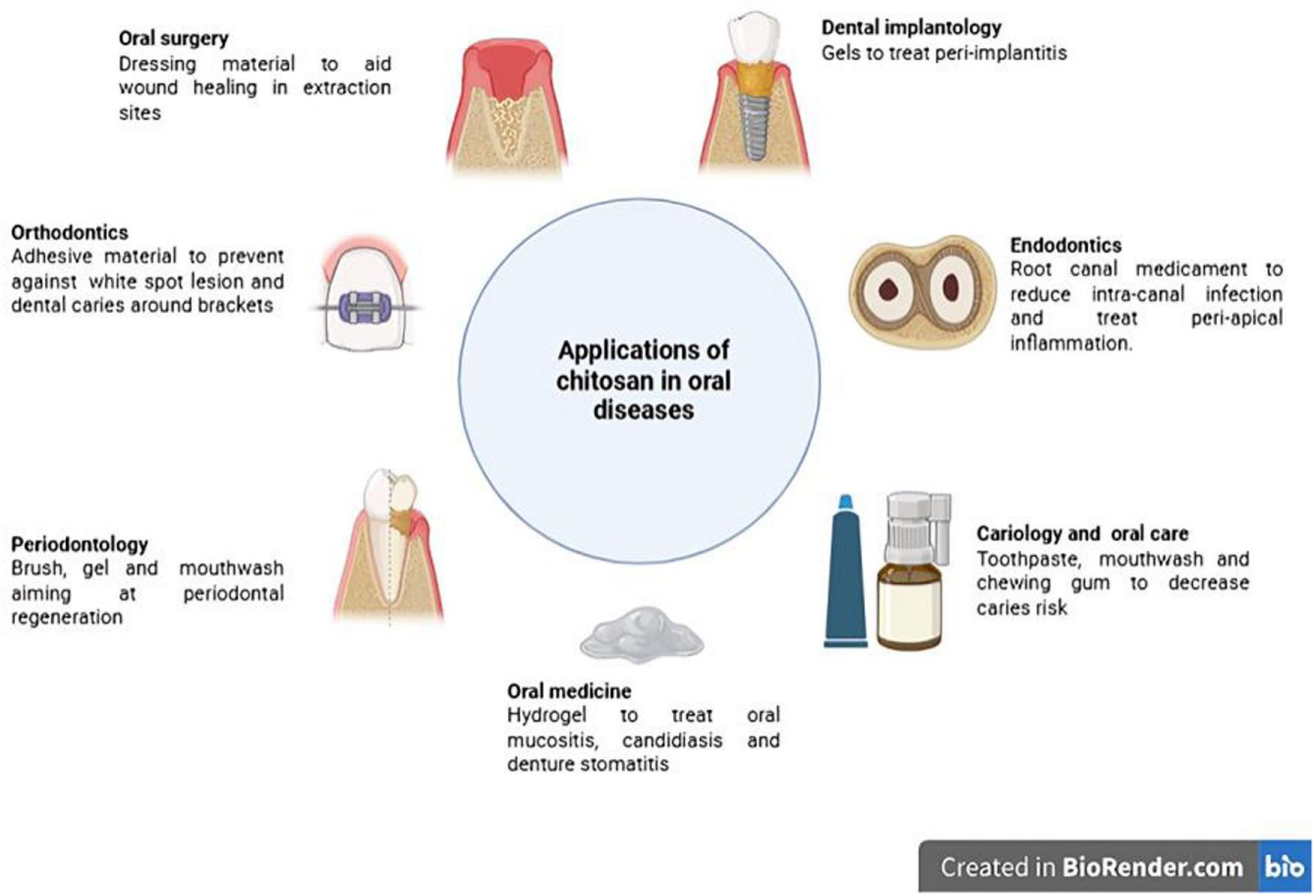
Applications of chitosan in stomatology.

This article reviewed the applications of chitosan in clinical human studies over the last ten years related to oral health and diseases. Chitosan has been used in nearly all the clinical aspects of dentistry and oral health. Due to the vast heterogeneity of the measured exposures and outcomes, the critical appraisal of the included articles will be presented according to their field of use. Figure 3 summarizes these different applications.

### Oral surgery

The findings were heterogenous. For example, Thakkar et al. compared chitosan with low-level laser therapy (LLLT) with controls in 87 post-extraction primary molars from 29 participants. Chitosan decreased pain, but there was no significant difference in the groups' bleeding times. Additionally, chitosan was the preferred intervention for the children and their parents. In the case that the diode laser is unavailable, the authors came to the conclusion that chitosan can be a very reliable, efficient, and affordable alternative (25). To achieve early wound healing and hemostasis at palatal donor sites in patients having free gingival graft (FGG) surgery, Tadav et al. employed chitosan-based dressings (CD). Despite not reaching statistical significance, they found that CD aided the wound healing process and boosted re-epithelialization. Additionally, it was discovered that CD considerably decreased bleeding issues, although it had no effect on pain levels (26).

The Bexident Post (BP) topical gel, which contained chitosan, dexpanthenol, allantoin, and chlorhexidine, was compared to bicarbonate. In patients having oral surgery, the BP gel outperformed bicarbonate in reducing pain and inflammation, lowering the need for analgesics, and promoting improved cicatrization (27). In another study, the same gel utilized vs. a placebo used for pain and inflammation control after third molar surgery. The gel significantly reduced postoperative pain, trismus and signs of inflammation (2). Zorilla et al. evaluated the effects of chlorhexidine-chitosan, chlorhexidine, hyaluronic acid gels and neutral water-based gel (control) on microbiologic colonization of the suture threads. Patients who received treatment with chlorhexidine-chitosan had higher healing rates than those who received hyaluronic acid or a placebo gel. Nevertheless, none of the bio-adhesive gels tested produced advantageous decreases in the bacterial/fungal populations in the microbiological investigations (6).

Hemcon Dental Dressing (HDD) based on chitosan, was applied to 33 individuals with two or more surgical sites who were taking oral anticoagulants (OAC). The HDD was demonstrated to be a clinically effective hemostatic dressing material that considerably reduced the amount of time that patients bled after minor oral surgical operations performed under local anesthetic. Additionally, compared to controls, patients who received the HDD showed better surgical wound healing (32). In addition, Radhakrishna used chitosan dressing in 54 patients on OACs. Chitosan-based dressings were more successful at reducing postoperative bleeding than cotton pressure packs (24). Furthermore, when chitosan is compared to Surgicel (a resorbable oxidized cellulose material) in liver cirrhosis patients, both were effective for controlling bleeding and were regarded as safe after tooth extraction (30).

To preserve the alveolar ridge after extraction, twelve patients (24 alveolar sockets) underwent electrospun chitosan/polyvinyl alcohol (CS/PVA) nanofibrous scaffolds, which were contrasted with secondary intention healing. After four months, the authors reported that, in comparison to the control group's results, the scaffold considerably reduced alveolar bone loss both horizontally and vertically and increased bone density in alveolar sockets (28).

Pain, inflammation, trismus and swelling are the most common complications of oral surgeries. Dental extraction sites are usually left to heal by secondary intention. However, there is always a risk of inflammation, known as alveolitis or dry socket that delays wound healing and tissue regeneration (63, 64). Traditionally, zinc oxide eugenol pastes were used in surgical sites to aid in healing. The other effective dressings include resorbable collagen and platelet rich fibrin (65, 66).

Moreover, patients on anticoagulants or those who have some impairment in their liver function, are at higher risk of extensive bleeding after oral surgeries. Control of bleeding in dental extraction sites could be challenging in these kinds of patients (67, 68). Therefore, dentists and oral surgeons tend to use wound dressings at a higher rate in these patients compared to healthy individuals. Utilization of local hemostatic agents such as absorbable gelatin sponge, oxidized cellulose and chitosan-based dressings may help in post-operative extraction site management in these kinds of patients with higher risk (69, 70).

Based on the studies included in this review, it can be concluded that chitosan can be used in oral surgery sites in a safe and effective manner. Chitosan-containing materials have shown some potential in oral surgery, including the management of extraction sites, due to their biocompatibility, hemostatic properties, antimicrobial activity, and ability to promote tissue regeneration. These attributes can facilitate faster healing, reduce the risk of infection, and improve patient comfort. Compared to standard treatments—such as saline rinses, gauze packing, and conventional wound dressings—chitosan-based materials may offer additional benefits, including enhanced clot stability, reduced inflammation, and improved tissue regeneration.

### Dental implantology

Mayer et al. used chitosan brush for 106 peri-implantitis cases compared to conventional curettage. Probing depth and bleeding on probing were significantly different between the groups at 6 and 12 months of follow-up. Additionally, they found that the mechanical phase's supplementary use of local antiseptics and anti-inflammatories was favorably correlated with the reduction of inflammation and the reattachment of connective tissue (12). In addition, when chitosan brush was utilized for professional removal of plaque in 15 patients with mild peri-implantitis, plaque index, bleeding on probing and periodontal pocket depth, significant results were documented. The authors stated that chitosan brushes may be a dependable tool for properly applied plaque cleaning and the alleviation of clinical symptoms associated with the early phases of peri-implant inflammation (15). In another study with 24 peri-implantitis sites, bleeding on probing was significantly different after 2 and 4 weeks between the groups (10). In contrast, chitosan brush compared to titanium curettes in three studies, revealed similar results for the two groups (11, 13, 42).

Peri-implantitis is the inflammation of the soft and hard tissue around dental implants and may eventually lead to implant failure if left untreated. Standard non-surgical treatment options for peri-implantitis include mechanical debridement, application of local or systemic antibiotics or antiseptics (e.g., chlorhexidine) to reduce bacterial load and, in some cases, laser therapy with varying evidence (71–73).

The use of a chitosan brush in the treatment of peri-implantitis is an emerging approach that leverages the antimicrobial and biocompatible properties of chitosan. Chitosan brushes can effectively reduce bacterial load and biofilm formation around implants, potentially aiding in the management of peri-implantitis.

Compared to standard treatments, such as mechanical debridement with curettes, ultrasonic scalers, or surgical interventions, chitosan brushes offer a minimally invasive, biocompatible option that may enhance plaque control and reduce inflammation. However, current evidence is limited, and more high-quality clinical trials are needed to establish their efficacy definitively.

### Periodontology

Findings of the studies were various. Hussain et al. used oscillating chitosan brush in periodontitis patients compared to conventional curettes. They reported a better and significant combined outcome of BOP and PPD ≤ 4 mm in the test group (45).

Two studies used a mixture of chitosan and antibiotics. Nano-structured doxycycline/chitosan mixture was used in 150 patients with moderate to chronic periodontitis and compared with doxycycline only and control groups. Both treatment groups had significant but similar effects in improving the measured parameters compared with the control (46). In another study, chitosan 2% gel containing free simvastatin and simvastatin microsponge were used in 24 patients with periodontal pockets. Chitosan gels containing simvastatin microsponges exhibited significant reduction in both PPD and CAL compared to those prepared with free simvastatin (48). This might be due to hydrogen bond formation between microsponges encapsulating simvastatinand chitosan, making them good carriers for local periodontitis therapy.

Chitosan was used as a coating material with photosensitizer nanoparticles in antimicrobial photodynamic therapy (aPDT) and applied in 40 periodontal pockets to evaluate its subgingival bacterial suppression capacity. Results of the colony count was significant, while the PPD difference was not significant (44). When a membrane of chitosan and nano-hydroxyapatite was utilized in 22 gingival recession sites and compared with amnion membrane, significant intragroup but similar intergroup results were found (47).

The main treatment options of periodontitis and periodontal defects are classified as surgical and non-surgical. The primary aims of non-surgical therapy are to control infection, reduce inflammation, and slow down the disease progression (74). The list of standard non-surgical treatment alternatives includes professional dental plaque removal (scaling) and root planning, adjunctive use of antimicrobial agents (like chlorhexidine mouthwash and gels), and the usage of local delivery systems (such as doxycycline gel and chlorhexidine chips) directly into periodontal pockets (75, 76). In the case of peri-implantitis, the same treatment modalities adapted to implants can be deployed (40, 76, 77).

Based on the literature reviewed in this article, chitosan brush, chitosan-antibiotic mixture, chitosan as adjunct to laser therapy and chitosan nanoparticles can be used in periodontitis cases with some predictable results. Chitosan-containing materials have demonstrated some promising antimicrobial, anti-inflammatory, and tissue regenerative properties that could be beneficial in the management of periodontitis. These properties may help in reducing bacterial load, promoting healing of periodontal tissues, and potentially improving clinical outcomes. While preliminary evidence suggests that chitosan-containing materials could enhance periodontal therapy outcomes, current data are insufficient to replace or significantly alter established treatment protocols.

### Oral medicine

Liberato da Silva used chitosan hydrogel containing 0.05% nano encapsulated imiquimod in 49 subjects with actinic cheilitis. The test group exhibited significantly higher rates of clinical improvement compared to the placebo (22). Moreover, chitosan nanogel and doxepin/chitosan nanogel were compared to doxepin mouthwash and diphenhydramine + aluminum-magnesium mouthwash in 60 patients with chemotherapy-induced oral mucositis. After two weeks of treatment, both lesion size and pain severity were significantly reduced in the treatment groups. Authors stated that chitosan-based doxepin nano-formulation might be a promising alternative for routine treatments of oral mucositis (23). In a recent clinical trial, miconazole-loaded chitosan nanoparticles were compared to miconazole to treat 80 diabetic patients with symptomatic oral candidiasis. Clinical improvement and colony number results, but not antifungal efficacy was significantly different between the two treatment types (49). Furthermore, when a mixture of topical chitosan nanogel and probiotic mixture was compared to an oral rinse control group to treat recurrent aphthous stomatitis among 60 patients, both lesion size and pain severity were significantly reduced in the chitosan group (50).

Three studies were related to the management of denture stomatitis (DS). Chitosan-curcuminoid (CHI-CUR) mouthwash was compared to chlorhexidine. Complete healing of erythematous lesions, but not the number of candidal colonies, was significantly different between the treatment groups after two weeks. The results showed that CHI-CUR mouthwash without alcohol could be a safe and effective topical treatment option for generalized or candida-related DS (52). Among 40 patients with denture stomatitis, in comparison to the normal nystatin treatment, chitosan solution dramatically reduced the number of blastospores and mycelia, the erythematous surface area, the burning sensation, and the time needed for clinical improvement. It was stated that chitosan is a viable option for use as an antifungal mouthwash due to its antifungal effectiveness and natural biocompatibility (53). When miconazole nitrate-loaded with chitosan nanoparticles and miconazole nitrate alone was used in 48 patients of DS, the chitosan containing treatment was significantly better in patients' pain scoring, colony count and anti-fungal potential. Authors concluded that miconazole-chitosan compound can be promoted as an efficient local treatment for denture-related candidiasis using less miconazole applied daily and less often than a commercial oral gel application (51).

Oral mucositis alone or concurrent with oral candidiasis is prevalent among elderly patients that have immunocompromising issues, have chronic medical conditions (mostly importantly diabetes), are on several different medications, undergo cancer treatment (chemotherapy), have poor oral hygiene or use dentures (78–80). The latter has a distinct cause and therefore called denture stomatitis (81).

Treatment of oral mucositis depends on the underlying cause. The standard treatment options include pain management through analgesics, use of mouthwashes with antimicrobial or anti-inflammatory properties (e.g., chlorhexidine) and using topical agents such as corticosteroids and protective gels (like sucralfate). In case of candidiasis, nystatin and clotrimazole are used (79, 82, 83).

Chitosan-containing materials show promising potential as adjunctive or alternative therapies for the management of oral mucositis and candidiasis due to their biocompatibility, antimicrobial properties, and ability to promote wound healing. The studies suggest that chitosan can reduce inflammation, enhance tissue regeneration, and inhibit fungal growth, making it a valuable component in oral care formulations.

However, in comparison to standard and established treatments—such as topical corticosteroids for mucositis and antifungal agents like nystatin or clotrimazole for candidiasis (84–86)—chitosan-based materials are generally considered supplementary rather than replacement options at this stage. While they offer advantages like reduced side effects and natural origin, more rigorous clinical trials are needed to firmly establish their efficacy, optimal formulations, and long-term safety.

### Restorative dentistry

Mishra et al. used chitosan modified glass ionomer cement (GIC) in 50 children and compared to chlorhexidine-cetrimide modified GIC. In the *in vivo* assessment, chitosan group showed higher and significant antibacterial activity (54). Thirty teeth with periapical changes were treated with native chitosan scaffold and blood clot (NCS + BC) scaffold, enzymatically modified chitosan and blood clot (EMCS + BC) scaffold and only blood clot (BC) scaffold as control. Although the EMCS + BC scaffold healed pulp better than the NCS + BC and BC groups after six months, but at one, three, and twelve months, there were no significant differences. In addition, EMCS + BC and NCS + BC promoted greater tooth sensitivity (55). Fifty-five single-rooted teeth with failed endodontic therapy were treated with ciprofloxacin hydrochloride (CIP) encapsulated in PLGA nanoparticles coated with chitosan (CIP-CS-PLGA-NPs) compared to free CIP incorporated in Pluronic®. Healing percentage, bacterial count and biofilm inhibition percentage were significant between the groups. The authors concluded that the chitosan group was the most effective treatment for recurrent *E. Faecalis* infections in endodontics, as evidenced by the highest percentage of biofilm inhibition and overall bacterial decrease. Furthermore, the antibiotic was successfully delivered by nanocarriers deep within the root canal, maintaining its effectiveness to eliminate the persistent *E. Faecalis* infection and subsequent biofilm formation (56). Nasr et al. treated 60 patients with necrotic mandibular premolars with 3% chitosan nanoparticles (CNPs), 2% chlorhexidine (CHX), CHX/CNPs and 5.25% Sodium hypochlorite (NaOCl) in a single-visit endodontic treatment. Anaerobic bacteria isolated from necrotic mandibular premolars were considerably more susceptible to the effects of CNPs and their combination with CHX than to either CHX or NaOCl alone. Compared to either CHX or NaOCl, the combination of CNPs and CNPs/CHX resulted in much less post-operative pain (57). Final disinfection using low power diode laser, final rinse using 0.2% nano chitosan irrigation and 1.25% sodium hypochlorite irrigating solution & 17% EDTA only as control group were used as root canal irrigation solutions in 45 patients having necrotic mature anterior teeth with periapical lesions. Although nano chitosan had the highest percentage of cases regaining sensitivity, the difference was not significant (58).

The standard intra-canal medications used in endodontics primarily serve to disinfect the root canal system, eliminate bacterial infection, and promote healing. The commonly used intra-canal medicaments include calcium hydroxide, chlorhexidine, iodoform-based pastes (e.g., Ledermix, Vitapex), camphorated monochlorophenol (CMPC) and mineral trioxide aggregate (MTA) (87–89).

Calcium hydroxide remains the most used intra-canal medicament in endodontics due to its effectiveness, safety profile, and ease of use (90). The choice of medicament depends on the clinical situation, microbial considerations, and practitioner preference (91).

Due to the few studies in the field of endodontics reviewed in this article, it is difficult to draw a clear conclusion. The current evidence on the application of chitosan-containing materials in endodontics is primarily from *in vitro* and few preliminary clinical studies. Therefore, more extensive, high-quality clinical trials are needed to establish their definitive role.

### Cariology and oral care

Three studies used chitosan in toothpastes conducted by the same group of researchers. The studies had the same sample size of 610 children of 4–8-year-olds followed-up for two years. Chitosan containing HAF1000 and HAF1450 toothpastes significantly reduced mean salivary pH value and cariogenic bacterial count over two years follow-up when compared to conventional toothpastes (33). Again, chitosan-containing toothpaste significantly reduced the enamel caries activity. For the dentinal lesions, although the chitosan containing toothpaste performed better than conventional monofluoridated toothpaste, the difference was not significant (35). In children using chitosan-containing toothpaste, the relative risk of caries was 39% and 29% lower in primary and permanent dentitions respectively compared to monofluoridated toothpastes (36).

Santos et al. used 2.5% chitosan solution or distilled water after selective caries removal in dentinal lesions. The number of *Streptococcus Mutans* was significantly reduced following chitosan treatment of the dentin. However, no discernible variations were seen between retention and secondary caries (34). Khamverdi compared chitosan chewing gum compared to placebo gum among young adults. The difference of salivary *Streptococcus Mutans* counts, unlike mean salivary pH, was significant among the groups (37).

Dental caries is an infectious disease characterized by progressive destruction of the dental hard tissue structure. Many modifiable and non-modifiable risk factors affect the initiation and progression of dental caries (92–94). The standard options to prevent and treat dental caries include a combination of behavioral, preventive, and restorative strategies. Fluoridated toothpastes account for the global decrease in dental caries prevalence (95).

Laboratory studies suggest that chitosan can inhibit cariogenic bacteria such as *Streptococcus Mutans* and may enhance the remineralization process, making it a valuable adjunct in caries management (96–98). The incorporation of chitosan into dental products began in the early 2000s, driven by research into its antimicrobial effects against oral pathogens (1, 99). Biomimetic hydroxyapatite and fluoride toothpaste may benefit children with active primary dentition caries lesions. The use of these kinds of toothpaste may reduce caries increment in children more than traditional fluoridated toothpaste. While not as widespread as fluoride or triclosan, the use of chitosan in toothpastes has gained interest due to its natural origin and antimicrobial properties, representing a promising avenue for future oral health products.

### Orthodontics

Farzanegan et al. bonded brackets with conventional adhesives containing 1% chitosan nanoparticles (NPs) and 1% TiO_2_NPs and compared them to conventional orthodontic adhesives aiming to prevent white spot lesions and dental caries. At one day, two months, and six months, there was a noticeable decrease in the experimental group's *Streptococcus Mutans* numbers as compared to the controls (59). In another study, three mouthwashes were compared in 45 orthodontic patients: colloidal chitosan-silver nanoparticles-fluoride nanocomposite (CCAgNPF), chlorhexidine 0.2% and a combination of the two. All mouthwashes reduced the salivary *Streptococcus Mutans*, however, no significant difference was found between the efficacy of CCAgNPF and chlorhexidine. The best and significant results were achieved by the combination of CCAgNPF and chlorhexidine mouthwashes (61). Chitosan-based varnish compared to chlorhexidine-fluoride varnish for brackets in 20 subjects with 200 teeth. In patients receiving fixed orthodontic therapy, the plaque pH was neutral over the course of six weeks, but the bacterial count was decreased by both chitosan-based varnish and chlorhexidine-fluoride varnish (60). Hasriati et al. compared the effect of 1% chitosan, 0.2% chlorhexidine digluconate and Aquadest in 30 patients with orthodontic mini screws. Although there was no significant difference between the treatment groups, chitosan and chlorhexidine both demonstrated antibacterial activity, lowering the overall bacterial count surrounding orthodontic micro screws (62).

Both fixed and removable orthodontic appliances may increase the risk of dental caries through extra plaque accumulation around wire and brackets. Moreover, gingivitis is another possible outcome (100, 101). To overcome these problems, and apart from regular oral hygiene measures, generally antimicrobial mouth rinses (such as chlorhexidine), fluoride varnishes/gels and, resin-based sealants are used (102). According to the studies reviewed, chitosan mouthwash is comparable with chlorhexidine especially in the reduction of bacterial load and, their mixture could produce even better results. They may offer benefits such as reducing plaque accumulation around orthodontic appliances, minimizing the risk of white spot lesions, and promoting tissue healing.

## Limitations

This review gathered some important strength points, most importantly the systematic and reproducible search strategy aiming to prevent against the most common bias of narrative reviews which is subjectivity. However, there were some other limitations that should be recognized. Direct comparisons and meta-analysis were difficult due to the variation in study designs, sample sizes, chitosan formulations, concentrations, and outcome measures among the 41 clinical studies that were included. The generalizability and long-term safety evaluation of chitosan-based therapies may be limited by the comparatively small sample numbers and brief follow-up periods of many research. Furthermore, there was significant diversity in control groups and treatment regimens throughout dental specialties. The predominance of preliminary or pilot studies in some areas, particularly endodontics and restorative dentistry, limits the strength of conclusions that can be drawn about chitosan's clinical efficacy compared to established treatments.

## Conclusions

Chitosan has proven antimicrobial, biocompatibility, biodegradability, solubility and film-forming properties. These characteristics make chitosan an excellent candidate for biomedical applications. Chitosan, either alone or in conjunction with other materials, are used in many preclinical and clinical studies related to oral health disease. There is a growing body of research in these areas indicating its raising trends.

Chitosan-containing materials are promising adjuncts in oral surgical procedures, particularly for promoting healing and controlling bleeding in post-extraction surgical sites.

Concerning periodontology and dental implantology, chitosan brush, mouthwash and gels can be used successfully to help regeneration of periodontal defects or reduce inflammation severity in peri-implantitis. Moreover, chitosan-containing materials hold promises as supportive agents in the treatment of oral mucositis and candidiasis, potentially enhancing healing and infection control.

Using chitosan in conservative dentistry and endodontics is still in its early stages. Chitosan-containing materials hold potential as adjunctive or alternative intracanal medicaments in endodontics, especially given their natural origin and multifunctional properties. Nonetheless, they are not yet considered replacements for established treatments like calcium hydroxide or chlorhexidine.

In terms of oral care and cariology, compared to standard treatments—such as fluoride application, sealants, and restorative procedures—chitosan-based agents are still largely in the experimental or early clinical research stages. They offer advantages like natural origin and multifunctionality especially when used as adjuncts to fluoride in toothpastes.

In orthodontic patients with a higher risk of developing dental caries and white spot lesions, preliminary studies suggest that chitosan-based varnish and adhesives could enhance oral health outcomes especially for controlling plaque and reducing demineralization. However, current evidence is limited.

In general, chitosan-containing materials show potential and useful characteristics as supportive and adjunctive agents when compared to established preventive and treatment measures. At this stage, their utilization in human clinical trials should be viewed as promising supplementary and adjuncts pending further validation through comprehensive clinical trials to confirm their comparative effectiveness and standardized application protocols.
