# Correction: Applications of chitosan in oral health and diseases

**DOI:** 10.3389/froh.2025.1730476

**Published:** 2025-11-04

**Authors:** Balen Hamid Qadir, Mohammed Khalid Mahmood, Tara Ali Rasheed, Kawan Salah Othman, Mohammed Abdalla Mahmood, Mohammed Aso Abdulghafor, Handren Ameer Kurda, Zana Fuad Noori, Botan Barzan Tahir, Mohammed Taib Fatih, Hevi Nihad Mohammed Fadhil, Vincent Romao, Arthur Falguiere, Romain Lan

**Affiliations:** 1 Department of Dentistry, Komar University of Science and Technology, Sulaymaniyah, Kurdistan, Iraq; 2 Faculty of Medical and Paramedical Sciences, French National Center of Scientific Research (CNRS), French Blood Establishment (EFS), Bio-Cultural Anthropology, Law, Ethics and Health (ADES), Aix-Marseille University, Marseille, France; 3 College of Dentistry, University of Sulaimani, Sulaimani, Kurdistan, Iraq; 4 College of Dentistry, The American University of Iraq—Sulaimani, Sulaymaniyah, Iraq; 5 Oral Surgery Department, Timone University Hospital, Marseille, Provence-Alpes-Côte d'Azur, France

**Keywords:** chitosan, dentistry, dental material, natural polysaccharide, dental health, marine drugs, oral health

Affiliation Oral Surgery Department, Timone University Hospital, Marseille, Provence-Alpes-Côte d’Azur, France was omitted for author Romain Lan. This affiliation has now been added for author Romain Lan.

The original version of this article has been updated.

